# Prevalence and determinants of activity of daily living and instrumental activity of daily living among elderly in India

**DOI:** 10.1186/s12877-021-02659-z

**Published:** 2022-01-19

**Authors:** Shekhar Chauhan, Shubham Kumar, Rupam Bharti, Ratna Patel

**Affiliations:** 1 Department of Family and Generations, International Institute for Population Sciences, Mumbai, India; 2 Department of Mathematical Demography & Statistics, International Institute for Population Sciences, Mumbai, India; 3 District Information and data Manager (DIDM)- Monitoring, Evaluation, & Learning, Social & Economic Empowerment, Jaipur, Rajasthan 302015 India; 4 Department of Public Health and Mortality Studies, International Institute for Population Sciences, Mumbai, India

**Keywords:** ADL, IADL, Elderly, India

## Abstract

**Background:**

The increase in life expectancy has proliferated the number of elderly and subsequently increased the prevalence of disability among the elderly. This study assesses the prevalence of Activity of Daily Living (ADL) and Instrumental Activity of Daily Living (IADL) and analyzes determinants of ADL and IADL among elderly aged 60 and over living in India.

**Methods:**

The study utilized the Longitudinal Ageing Study in India (LASI, 2017–18) data, and information was sought from 31,464 elderly aged 60 years and above. An index of ADL and IADL was created on a scale of three levels, exhibiting no, moderate, or severe levels of ADL/IADL disability. Multinomial logistic regression was used to determine the effect of socio-demographic parameters on ADL and IADL disability among the elderly.

**Results:**

Around 3% of the elderly reported severe ADL disability, and 6% elderly reported severe IADL disability. Elderly who were not involved in any physical activity than their counterparts were more likely to report severe ADL (RRR = 2.68, C.I. = 1.66–4.32) and severe IADL (RRR = 2.70, C.I. = 1.98–3.67) than no ADL and no IADL, respectively.

**Conclusion:**

Amidst the study finding, the study emphasizes the importance of setting-up of geriatric care centers in rural and urban areas. It would be feasible to provide geriatric care under the umbrella of already functioning government health facilities in different parts of the country. Community interventions earmarking the elderly with a focus on physical activity, specifically based in group physical exercise and implemented through existing networks, are rewarding for the elderly.

## Background

Globally, the life expectancy at birth has increased from 66.5 years in 2000 to 72 years in 2016 (Patel et al., 2019). On the back of improvements in the educational system, health facilities, and life expectancy, the percentage of elderly in India had risen from 5.3% in 1971 to 5.7% in 1981 and further from 6% in 1991 to 8% in 2011 [1]. Furthermore, the decline in fertility levels and increase in life expectancy have led to a rise in the absolute number of elderly in India [2]. Ageing across the countries has been increased for more than 35 years on policy discourse [3]. However, the focus across countries was on demographic transition instead of ageing [4]. The developed countries have moved ahead in providing both healthy and quality life to their citizens than developing countries [5].

Over 1 billion (15%) individuals worldwide have experienced one or more disability conditions. The global trends among the ageing population and the risk of disability lead to a higher disabled population [6]. Disability is commonly defined as a difficulty in performing everyday activities necessary for independent living, such as basic Activities of Daily Living (ADL) and Instrumental Activities of Daily Living (IADL) [7]. The higher disability rates result from health risks across various diseases, chronic illness, and injury [1]. Globally, a person with disabilities faces many hindrances in their life. It includes attitudinal, environmental, and institutional barriers which prevent their full participation in any aspects of life [6]. Disability results from health problems and interactions between health conditions, activity and participation, and environmental and personal factors [8]. Agenda 2030 for Sustainable Development pledges that no one will be left behind. Its integral part is to promote and protect older adults’ rights and dignity and facilitate their full support in society [9].

In the twenty-first century, the low and middle-income countries have experienced an upward shift in life expectancy [6]. Thus, it leads to an increase in longevity and leads to multiple co-morbidity conditions, commonly referred to as ‘multimorbidity condition,’ and has become more common among the older adult population [10]. High- and low-income countries show that older adults are at increased risk for multiple chronic diseases [11–13].Longevity results in chronic diseases that affect functionality, compromising the ability to pursue the daily routine and creating a need for assistance. Several factors have been linked to the onset of functional disability among older adults. Socioeconomic status is strongly associated with the prevalence of morbidity where the socioeconomic status is measured through education [14, 15], occupation [16], income [17], or whether it is found to be an area-based deprivation [18]. A study focused on East-Mediterranean countries, a review of 26 studies on multimorbidity, resulted in a low level of education, low income, and unemployment associated with the higher prevalence of multimorbidity among older adults [19]. It is also associated with adverse health outcomes like reduced physical function [20], poor quality of life [21], and self-rated health as poor [22], and mortality [23]. Morbidity among older adults is a likely cause of functional disability, as discussed above.

Many studies were conducted for older adults, which measured their functional performance through self-reported ADL andIADL based on their daily activities. Examining functional disability through ADL and IADL provides a better insight as these two indicators cover a range of disabilities. However, unfortunately, the tools do not provide a clear picture of the actual functional capacity of an older person [24]. Thus, in this study, we have tried to examine various indicators among older adults in India. The indicators include- gender, age, education, marital status, living arrangement, place of residence, wealth index, health insurance, use of tobacco (self-rated health), and physical activity performed by the older adults.

## Methods

### Ethical considerations

This study is based on secondary data available in the public domain. Anyone can access the data without any legal or ethical considerations. Therefore, there is no ethical approval required for this study as this study did not involve human or animal participants directly. However, the Indian Council of Medical Research (ICMR) provided the ethical approval for conducting the Longitudinal Ageing Study in India (LASI) survey. Also, informed consent was provided to the participants before undertaking the survey. To maximize the cooperation of the sampled households (HHs) and individuals, participants were provided with information brochures explaining the purpose of the survey, ways of protecting their privacy, and the safety of the health assessments as part of the ethics protocols. As per ethics protocols, consent forms were administered to each HH and age-eligible individual.

#### Data

We used the data from the Longitudinal Ageing Study of India (LASI), Wave-1, a longitudinal survey of the older men and women age 45 years and above in India [25]. The LASI is the first-ever survey in India that provides comprehensive data on health, economics, and social determinants and the consequences of population ageing in all 35 states (except Sikkim) and union territories in India. The survey has used a multistage stratified area probability cluster sampling design to cover an appropriate sample of the elderly. LASI is a nationally representative survey of 72,250 older adults and above, which plan for every 2 years for the next 25 years with refreshment samples for attrition due to death, dislocation, non-contact, and refusal. Our study was concerned with 31,464 elderly aged 60 years and above.

#### ADL disability and IADL disability

Activities of daily living (ADL) and instrumental activities of daily living (IADL) disability were self-reported scores of functional limitations recorded over more than 3 months. These functional problems that occurred in the last less than 3 months were excluded from the study. The ADL scale was considered from five indicators: bathing, dressing, mobility, feeding, and toileting. Further, ADL has been categorized into three categories as “severe ADL disability,” “moderate ADL disability,” and “No ADL disability.” Severe ADL ability is considered as those elderly who were not able to do in any of five activities, moderate ADL disability included those elderly who could not function in less than five activities, and no ADL disability included elderly who were able to perform in all five activities [26].

Further, the IADL scale [27] covered seven instrumental activities: preparing a hot meal (cooking and serving), shopping for groceries, making telephone calls, taking medications, doing work around the house or garden, managing money, such as paying bills and keeping track of expenses and getting around or finding an address in an unfamiliar place. Similarly, the IADL disability has been categorized into three categories as “severe IADL disability,” “moderate IADL disability,” and “no IADL disability.” Severe IADL disability includes those elderly who could not do any of seven activities; moderate IADL disability includes those elderly who could function less than seven activities. No IADL disability had to those elderly who were able to perform in all seven activities.

#### Covariates

The covariates included sex (male and female); age (60–69 and 70 years and above); marital status (Currently married; never married; education (No education; below primary; primary; secondary; higher); living arrangements (living alone, with spouse and with others), place of residence (rural and urban); wealth index (poorest, poorer, middle, richer and richest); covered with health insurance (yes and no); use of tobacco (yes and no); self-rated health (poor and good) and physical activities (yes and no).

#### Statistical analysis

Data were analyzed using STATA version 16. Descriptive statistics was carried out to estimate the proportion of ADL and IADL by socio-demographic variables among the elderly. Bivariate analysis was adopted to estimate the prevalence of ADL and IADL disability with the level of significance. In addition to that, the chi-square test was used to check the level of significance. Further, multinomial logistic regression was used to determine the effect of socio-demographic parameters on ADL and IADL disability among the elderly. Multinomial logistic regression is used in categorical dependent variable/s found with two or more unordered levels. The outcome of multinomial logistic regression comes in terms of relative risk ratio (RRR), the probability of choosing one outcome category over the baseline category. The equation of multinomial logistic regression is;

$$RRR=\frac{P\left(y=1\right|x+1\left)P/\left(y= base\ category\right|x+1\right)}{P\left(y=1\right|x\Big)/P\left(y= base\ category|x\right)}$$

Where RRR is the relative risk ratio, and P is the probability of occurrence. If the RRR is equal to 1, then the association between the response variable to the exposed group are unlikely to exist, when RRR > 1 then increases the risk of response variable among the exposed group and when RRR < 1 then decreases the risk of response variable among the exposed group. All methods were performed in accordance with the relevant guidelines and regulations.

## Results

**Fig. 1:**
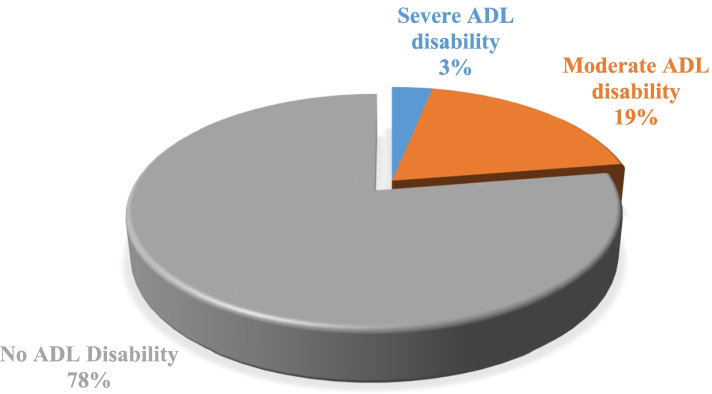
Prevalence of ADL among elderly in India. Legend: No ADL disability, Moderate ADL disability, & Severe ADL disability

Figure 1 depicts the prevalence of ADL among the elderly. More than two-thirds (78%) of the elderly did not report any ADL disability. Around one-fifth (19%) of the elderly had moderate ADL disability, and the remaining 3% had severe ADL disability.

**Fig. 2:**
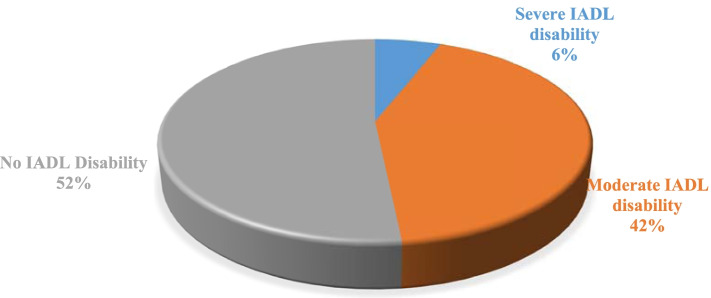
Prevalence of IADL among elderly in India. Legend: No IADL disability, Moderate IADL disability, & Severe IADL disability

Figure 2 depicts the prevalence of IADL among the elderly. More than half (52%) of the elderly did not report any IADL disability. Around two-fifth (42%) of the elderly had moderate IADL disability, and the remaining 6% had severe IADL disability.

Table 1 depicts the background characteristics of the elderly population. Nearly 47.5% of the sample consisted of male elderly, and the remaining (52.5) consisted of female elderly. Almost 62% of the elderly were married, and more than one-third (37.2%) were divorced/separated/widowed. More than half of the elderly (56.5%) had no education. Nearly 6% were living alone, and three-fifths (60.9%) were living with a spouse. Around four-fifths (81.8%) of the elderly were not covered by any health insurance.

**Table 1:** Characteristics of total sample of elderly by sociodemographic parameters in India

		N	%
Sex	Male	14,931	47.5
	Female	16,533	52.6
Age	60–69	18,410	58.5
	70+	13,054	41.5
Marital status	Currently married	19,536	62.1
	never married	225	0.7
	divorced/separated/deserted	11,703	37.2
Education	No Education	17,782	56.5
	Below primary	3598	11.4
	Primary	3520	11.2
	Secondary	5285	16.8
	Higher	1278	4.1
Living arrangement	Living alone	1787	5.7
	With spouse	19,176	60.9
	With others	10,501	33.4
Place of residence	Rural	22,196	70.6
	Urban	9268	29.5
Wealth Index	Poorest	6829	21.7
	Poorer	6831	21.7
	Middle	6590	21.0
	Richer	6038	19.2
	Richest	5175	16.5
Covered with health Insurance	Yes	5685	18.2
	No	25,477	81.8
Use of tobacco	No	18,665	59.8
	Yes	12,539	40.2
Self-rated health	Poor	4630	15.0
	Good	26,181	85.0
Physical activity	Yes	9704	31.1
	No	21,494	68.9
Total		31,464	100

Table 2 depicts the prevalence of ADL and IADL among the elderly by various background characteristics. These results are from cross-tabulation and are presented with a *p*-value. A higher percentage of female elderly had severe ADL disability (3.5% vs 2.8%) and severe IADL disability (7.5% vs. 4.2%) than their male counterparts. Similarly, a higher percentage of elderly aged 70+ had severe ADL disability (5.7& vs 1.4%) and IADL disability (11.1% vs 2.3%) than elderly who were 60–69 years of age. Around 6.5% of the never-married elderly had severe ADL disability. A higher percentage of uneducated elderly had severe ADL disability (2.9% vs 1.9%) and severe IADL disability (4.6% vs 1.6%) than elderly who had higher education. Furthermore, severe ADL and IADL disabilities were more prominent among rural elderly, poorest elderly, those who were not covered by health insurance, who had poor self-rated health and were not involved in any physical activity than their respective counterparts.

**Table 2:** Prevalence of ADL disability (severe, moderate, and no ADL disability) and IADL disability (severe, moderate, and no IADL disability) among elderly by sociodemographic parameters in India

		ADL Disability				IADL Disability			
		Severe ADL disability	Moderate ADL disability	No ADL Disability	*P*-value	Severe IADL disability	Moderate IADL disability	No IADL Disability	*P*-value
Sex	Male	2.8	16.8	80.4	0.000	4.2	34.6	61.2	0.000
	Female	3.5	21.6	74.9		7.5	49.4	43.1	
Age	60–69	1.4	15.2	83.4	0.000	2.3	38.5	59.1	0.000
	70+	5.7	25.1	69.1		11.1	48.0	41.0	
Marital status	Currently married	2.3	17.0	80.7	0.000	3.9	37.8	58.4	0.000
	Never married	6.5	13.3	80.2		9.4	37.1	53.6	
	Divorced/Separated/Deserted	4.6	23.2	72.3		9.3	50.3	40.5	
Education	No Education	3.9	21.2	75.0	0.000	8.1	48.6	43.3	0.000
	Below primary	2.9	24.8	72.4		4.6	44.5	50.9	
	Primary	2.5	16.1	81.4		3.8	33.8	62.5	
	Secondary	1.9	13.3	84.8		2.0	30.5	67.4	
	Higher	1.9	10.7	87.4		1.6	21.7	76.7	
Living arrangement	Living alone	2.5	23.6	73.9	0.000	5.7	53.8	40.5	0.000
	With spouse	2.3	16.9	80.8		3.8	37.6	58.6	
	With others	4.8	22.9	72.3		9.8	49.3	40.9	
Place of residence	Rural	3.3	20.0	76.7	0.245	6.8	44.8	48.4	0.000
	Urban	3.0	17.6	79.4		3.9	36.6	59.5	
Wealth Index	Poorest	4.1	20.4	75.5	0.245	7.6	42.8	49.7	
	Poorer	3.0	19.8	77.2		5.7	44.0	50.3	
	Middle	2.9	19.3	77.8		5.8	40.1	54.1	
	Richer	2.5	17.9	79.6		4.8	43.9	51.3	
	Richest	3.4	18.7	77.9		5.5	41.2	53.3	
Covered with health Insurance	Yes	2.3	15.5	82.2	0.000	3.9	39.5	56.6	0.000
	No	3.4	20.2	76.4		6.4	43.2	50.4	
Use of tobacco	No	3.4	19.0	77.7	0.003	6.2	43.2	50.7	0.000
	Yes	2.9	19.9	77.3		5.6	41.5	53.0	
Self-rated health	Poor	7.9	33.6	58.5	0.000	13.9	53.4	32.7	0.013
	Good	1.6	16.5	82.0		3.5	40.6	55.9	
Physical activity	Yes	0.9	13.5	85.6	0.000	1.7	37.1	61.2	0.000
	No	4.2	21.9	73.9		7.8	44.9	47.2	

Table 3 depicts the relative risk ratio computed from multinomial logistic regression for ADL and IADL among the elderly by various background characteristics. Multinomial logistic regression has two reference categories; the first reference category is the base outcome for ADL and IADL (no ADL/no IADL), and the second reference category is that of respective background variable (For ex. Male is the reference category for background variable named ‘sex’). Since multinomial logistic regression has two reference categories, the results are to be understood while taking both the reference category together. As compared to older men, older women were 1.25 times (RRR = 1.25, C.I. = 1.10–1.42) more likely to report moderate ADL disability than no ADL disability, whereas 1.70 times (RRR = 1.70, C.I. = 1.35–2.14) more likely to report severe IADL disability and 1.63 times (RRR = 1.63, C.I. = 1.45–1.84) moderate IADL disability than no IADL disability. Age is one of the strongest predictors of severe ADL and IADL among the elderly. Results found that higher educated elderly than uneducated elderly were less likely (RRR = 0.64, C.I. = 0.48–0.87) to report moderate ADL as compared to no ADL. Compared to the rural elderly, the urban elderly had a lower risk of reporting (RRR = 0.52, C.I. = 0.41–0.65) severe IADL than no IADL. Elderly who were not covered with health insurance than their counterparts were more likely to report ADL disability (RRR = 1.49, C.I. = 1.10–2.01) and IADL (RRR = 1.58, C.I. = 1.23–2.03) than no ADL disability and no IADL disability, respectively. Elderly consuming tobacco than their counterparts were less likely to report severe ADL (RRR = 0.74, C.I. = 0.56–0.98) than no ADL. Self-rated health is another significant predictor of ADL and IADL disability among the elderly. Elderly who reported good self-rated health than those who reported poor self-rated health were less likely to report severe ADL (RRR = 0.16, C.I. = 0.13–0.21) and severe IADL (RRR = 0.18, C.I. = 0.14–0.22) than no ADL and no IADL disability, respectively. Elderly who were not involved in any physical activity than their counterparts were more likely to report severe ADL (RRR = 2.68, C.I. = 1.66–4.32) and severe IADL (RRR = 2.70, C.I. = 1.98–3.67) than no ASL and no IADL, respectively.

**Table 3:** Multinomial regression analysis for ADL and IADL disability among elderly in India

		ADL Disability				IADL Disability			
		Severe ADL disability		Moderate ADL disability		Severe IADL disability		Moderate IADL disability	
		Relative risk ratio (RRR)	CI at 95%	Relative risk ratio (RRR)	CI at 95%	Relative risk ratio (RRR)	CI at 95%	Relative risk ratio (RRR)	CI at 95%
Sex	Male®								
	Female	0.90	0.68–1.18*	1.25***	1.10–1.42*	1.70***	1.35–2.14*	1.63***	1.45–1.84*
Age	60–69®								
	70+	3.16***	2.44–4.09*	1.64***	1.46–1.84*	4.76***	3.88–5.85*	1.58***	1.40–1.79*
Marital status	Currently married®								
	Never married	5.47**	1.26–13.73*	0.45**	0.23–0.88*	1.38	0.39–4.77*	0.79	0.46–1.34*
	Divorced/Separated/Deserted	2.34	0.84–6.46*	0.69**	0.47–1.00*	0.81	0.37–1.77*	0.96	0.67–1.34*
Education	No Education®								
	Below primary	1.04	0.63–1.70*	1.38***	1.15–1.64*	0.68	0.46–0.99*	0.91	0.79–1.03*
	Primary	0.73	0.49–1.07*	0.84**	0.71–1.00*	0.47***	0.34–0.65*	0.59***	0.52–0.68*
	Secondary	0.61**	0.40–0.91*	0.73***	0.59–0.89*	0.28***	0.20–0.41*	0.57***	0.45–0.72*
	Higher	0.59	0.29–1.17*	0.64***	0.48–0.87*	0.26***	0.13–0.50*	0.41***	0.29–0.59*
Living arrangement	Living alone®								
	With spouse	3.05**	0.98–9.53*	0.64**	0.42–0.96*	0.92	0.38–2.21*	0.73	0.50–1.06*
	With others	1.61**	0.97–2.69*	1.00	0.81–1.23*	1.86***	1.28–2.70*	1.01	0.82–1.23*
Place of residence	Rural®								
	Urban	1.05	0.79–1.39*	0.90	0.78–1.04*	0.52***	0.41–0.65*	0.76***	0.67–0.87*
Wealth Index	Poorest®								
	Poorer	0.70**	0.50–0.98*	0.97	0.82–1.12*	0.79	0.60–1.03*	1.07	0.94–1.20*
	Middle	0.65**	0.46–0.91*	0.97	0.83–1.13*	0.74**	0.55–1.00*	0.94	0.82–1.07*
	Richer	0.58***	0.41–0.82*	0.90	0.75–1.06*	0.71	0.53–0.94*	1.12	0.94–1.33*
	Richest	0.93	0.59–1.44*	1.00	0.82–1.21*	0.94	0.67–1.30*	1.11	0.93–1.31*
Covered with health Insurance	Yes®								
	No	1.49***	1.10–2.01*	1.35***	1.18–1.53*	1.58***	1.23–2.03*	1.15**	1.02–1.29*
Use of tobacco	No®								
	Yes	0.74**	0.56–0.98*	1.15**	1.03–1.28*	0.94	0.76–1.15*	1.08	0.98–1.18*
Self-rated health	Poor®								
	Good	0.16***	0.13–0.21*	0.39***	0.34–0.44*	0.18***	0.14–0.22*	0.48***	0.43–0.55*
Physical activity	Yes®								
	No	2.68***	1.66–4.32*	1.46***	1.27–1.67*	2.70***	1.98–3.67*	1.21***	1.09–1.35*

Note- No ADL/IADL Disability is considered as the base model, and® indicates the reference category. RRR of severe disability and moderate disability were calculated against no disability as the reference value

*** if *p* < 0.01; ** if *p* < 0.05; * if *p* < 0.1

## Discussion

Over the last few decades, India has witnessed a remarkable increase in life expectancy and a significant increase in the proportion of older adults [28]. Unfortunately, a striking proportion of older adults is more vulnerable to ageing, leading to poor well-being [29]. All this together leads to poor quality of life among the elderly. Therefore, understanding the determinants that affect the ADL and IADL is crucial in formulating the policy perspective. Hence, this study intends to determine the factors associated with ADL and IADL among the elderly in India and examine the prevalence of ADL and IADL among the Indian elderly. This study noted that almost 22% of older adults reported ADL, and 48% of older adults reported IADL. On the expected lines, a previous study in the Indian context also noted that a higher proportion of older adults reported IADL than ADL in India [30]. This is interesting to note that Patel et al. (2021) used decade-old BKPAI data [30]. In a study by Patel et al. (2021), almost 8% of older adults were reported not being independent for ADL, and another 57% of older adults were reported not being independent for IADL [30].

Female elderly were more likely to have the risk of ADL and IADL limitation than male elderly. Previous studies are in line with the finding of this study [1, 31–34]. Studies worldwide have also shown that the female gender is one of the risk factors for disability in old age [35, 36]. Female elderly are still neglected in terms of care with a minuscule focus on their health; it is due to gender-segregated behavioural activities in our society that makes females more vulnerable than males [34]. Researchers feel that gender discrimination in a male-dominated society like India makes females more vulnerable to the risk of disabilities [33]. Furthermore, women in India are more likely to ignore their health and are less likely to seek appropriate healthcare [37], which may further aggravate their risk of ADL and IADL [37, 38]. Also, gender inequalities in allocating resources like education, income, political voice, nutrition, and health-care, are very strongly associated with poor health and reduced well-being [39, 40]. A study noted that men were more likely to report needing help with cooking meals, doing laundry, and taking medicines. This has substantial weightage on why a higher percentage of older men report limitations with IADL than older women [41].

Age is another strongest predictor of poor ADL and IADL among the elderly. Despite having two categories in age (60–69 years and 70 and above), the importance of increasing age in highlighting the functional disability cannot be ruled out. The study found that the risk of severe ADL and IADL increases with an increase in age of the elderly. Almost all the research in the literature arena concord with this finding [30, 42–45]; however, few studies stated that onset of disability can be a reversible event or can reduce overtime during the ageing process [46, 47]. To corroborate with the findings of Hung et al. (2011) and Lin et al. (2012), it is imperative to be apprised of and address modifiable factors amalgamated with ADL and IADL [8]. A positive relationship between age and chronic disease suggests that chronic diseases among the elderly increase with their age [40]. Further, literature has established an association between chronic disease and ADL and IADL disability among the elderly [48, 49].

The study noticed that the risk of disability was lower among the elderly with higher education than their uneducated counterparts. The Association between functional disability and the education status of the elderly is also well established [50, 51]. Hu et al. (2005) believe that increased resource availability linked to higher education may ameliorate self-perception and decrease limitations with various health conditions [50]. The odds of severe IADL disability were lower among urban elderly than their rural counterparts. Previous studies agree with this study in finding that rural elderly tend to have a higher risk of IADL disability than their urban counterparts [44, 52]. The availability of better healthcare infrastructure in urban areas could be attributed to a lower risk of IADL disability among the urban elderly. In rural areas, the elderly depend more on family members or other people to manage their finances, payments, and purchases and avoid travelling to carry out these functions, leading to severe IADL among them [52]. Studies have noted that the elderly in urban areas have better access to healthcare, availability of logistic support in transportation, and better financial support as retirement benefits that keep them free from functional disabilities [49].

In reference to the elderly living alone, elderly living with spouses had a higher risk of severe ADL disability. This finding inculpates that elderly living alone tends to help themselves by carrying out work required for daily living; therefore, these elderly are less likely to report severe ADL disability than those who live with their spouse. Further, those with good self-rated health had a lower risk of reporting severe ADL and IADL related disabilities than those who reported poor self-rated health. Previous studies also highlighted that poor self-rated health affects limitations associated with ADL and IADL among the elderly [43, 53]. In connection with the possible relationship between self-rated health (SRH) and IADL, Tomioka, Karumatani, & Hosoi (2017) believe that older adults with better SRH may be more likely to engage in social activities that promote better outcomes for IADL among them [43].

The elderly who were not physically active had a higher risk of severe ADL and IADL disability than physically active ones. Studies have noted that physical activities improve ADL and IADL related disabilities among the elderly [8, 54]. Physical activity is a preventive and therapeutic factor that reduces the risk of physical and mental disorders and affects the maintenance of independence in everyday life [55]. The safeguarding effect of physical activity on ADL disability is an outcome of complex pathways and is likely to be multifactorial [56]. To put that in perspective, being physically active has been linked to reducing inflammation biomarkers which further avert chronic disease. Further physical activity may increase social interactions preventing depression; all these pathways combined may prevent disability among the elderly [56].

### Limitations and strengths of the study

The study is not free from some potential limitations. The foremost limitation is the self-reporting of data related to ADL and IADL. However, several previous studies measured ADL and IADL through self-reporting data only [30, 52, 54]. Furthermore, information related to self-rated health was also self-reported. The self-reporting of critical information may have led to some biases that could have affected the study findings. We are limited to the construction of ADL and IADL with five and seven indicators, respectively. However, Katz has constructed the ADL with the help of six indicators, namely, bathing, continence, dressing, mobility, feeding, and toileting. In our study, “continence” is absent while constructing the ADL due to the unavailability of information on “continence” in the data. Similarly, Lawton has developed the IADL with the help of eight indicators, but our study has included seven indicators only. While constructing IADL, “Laundry” was absent for the same reason as mentioned in ADL. Also, we could not establish causality between our study variables as the data were cross-sectional. However, such limitations do not comprise the results since the opted methodological procedures were enough to achieve the proposed objective. Despite the above limitations, the study has some considerable strengths too. The study is based on the latest data source that provides in-depth details about various parameters for the elderly in India. Furthermore, a pilot study was successfully carried out in 2010 to test the survey tools and protocols and understand how to strengthen the main survey process, i.e., the current survey. The data were collected through the Computer-Assisted Personal Interview (CAPI) technique, ensuring data quality through built-in checks in CAPI and real-time data monitoring with an automated data quality control protocol.

## Conclusion

Disability is the best quality of life indicator as it captures both diseased and non-diseased persons and hence provides an unambiguous assessment of well-being than traditional morbidity and mortality data [36]. There is growing evidence that female gender and increasing age of elderly are the two important risk factors of disability. This study also determined female gender and increasing age of the elderly as the important risk factor for severe ADL and IADL disability. Furthermore, education, place of residence, health insurance, self-rated health, and physical activity also significantly impact the prevalence of ADL and IADL disabilities among the elderly in India, as outlined in this study. Even though governments have started to plan for the well-being of their ageing society in some developed countries, there remains a ubiquitous need to raise awareness about the importance of population ageing in India. Based on the study finding, it is suggested to give proper attention to female elderly. Amidst the study finding, the study emphasizes the importance of setting-up of geriatric care centers in rural and urban areas. It would be feasible to provide geriatric care under the umbrella of already functioning government health facilities in different parts of the country. Promoting physical activity among the elderly through various channels would bring the desired result. Community interventions earmarking the elderly with a focus on physical activity, based explicitly on group physical exercise and implemented through existing networks, are rewarding for the elderly [57].

## Data Availability

The datasets generated and/or analysed during the current study are available with the International Institute for Population Sciences, Mumbai, India repository and could be accessed from the following link:https://iipsindia.ac.in/sites/default/files/LASI_DataRequestForm_0.pdf. Those who wish to download the data have to follow the above link. This link leads to a data request form designed by International Institute for Population Sciences. After completing the form, it should be mailed to: datacenter@iips.net for further processing. After successfully sending the mail, individual will receive the data in a reasonable time.

## Abbreviations

ADL: Activities of Daily Living
CAPI: Computer-Assisted Personal Interview
CI: Confidence Interval
IADL: Instrumental Activities of Daily Living
LASI: Longitudinal Ageing Study in India
RRR: Relative Risk Ratio
